# Potential Therapeutic Effects of Underground Parts of* Kalanchoe gastonis-bonnieri* on Benign Prostatic Hyperplasia

**DOI:** 10.1155/2019/6340757

**Published:** 2019-01-02

**Authors:** Antonio Palumbo, Livia Marques Casanova, Maria Fernanda Paresqui Corrêa, Nathalia Meireles Da Costa, Luiz Eurico Nasciutti, Sônia Soares Costa

**Affiliations:** ^1^Instituto de Ciências Biomédicas, Universidade Federal do Rio de Janeiro, 21941-902 Rio de Janeiro, RJ, Brazil; ^2^Instituto de Pesquisas de Produtos Naturais, Universidade Federal do Rio de Janeiro, 21941-902 Rio de Janeiro, RJ, Brazil; ^3^Programa de Carcinogênese Molecular, Centro de Pesquisas, Instituto Nacional do Câncer, 20231-050 Rio de Janeiro, RJ, Brazil

## Abstract

Benign Prostatic Hyperplasia (BPH) affects mainly older men. It is estimated to affect 50% of 51-60-year-old men and 70% of 61-70-year-old men. BPH is a nonmalignant proliferation of epithelial and stromal cells of the prostate gland regions. Despite the use of conventional pharmacological therapy, herbal medicines are used in BPH therapy, and several mechanisms of action have been suggested based on their complex chemical composition. Considering the ethnomedicinal uses of* Kalanchoe gastonis-bonnieri* (KGB), we evaluated the inhibitory effects on the proliferation of stromal cells from primary benign prostatic hyperplasia (BPH) of four different aqueous extracts from this plant: underground parts from specimens in flower (T1 treatment), leaves from specimens in flower (T2 treatment), and flowers (T3 treatment) and leaves from specimens not in flower (T4 treatment). T1, T2, T3, and T4 treatments at 250 *μ*g/ml for 72 hours inhibited BPH cells by 56.7%, 29.2%, 39.4%, and 13.5%, respectively, showing that the KGB underground parts extract (T1 treatment) was the most active. Our findings show that the extract of the KGB underground parts (150 and 250 *μ*g/ml) stimulates important changes in the BPH cells, modulating crucial processes such as proliferation, viability, and apoptosis. HPLC-DAD-MS/MS analysis provided a tentative identification of glycosylated syringic acid derivatives, glycosylated forms of volatile compounds, and lignans in this extract. Finally, these results suggest that there is a potential therapeutic use for KGB in BPH, which could improve the clinical management of the disease.

## 1. Introduction

Benign Prostatic Hyperplasia (BPH) is a nonmalignant proliferation of epithelial and stromal cells of the prostate gland, causing an enlargement of the gland that may or may not be associated with lower urinary tract symptoms (LUTS) which affect the quality of life [1–4].

BPH affects mainly older men; and the prevalence increases with age. BPH is estimated to affect 50% of 51-60-year-old men, and this number reaches 80 to 90% for men over 80 years old [2, 5, 6].

Two antagonistic phenomena are involved in maintaining the normal size of the prostate: the rate of cell proliferation and apoptosis (cell death). In normal tissue, these ratios are similar for both the epithelial and stromal cells. However, in BPH there is an imbalance where the cell proliferation rate increases considerably more than the rate of the apoptosis process [4, 7, 8]. There are evidences that androgens, estrogen, growth factors, and neurotransmitters may play an important role in the etiology of BPH [3]. Additionally, scientific and clinical studies have shown that an inflammatory process may also influence the onset of this disease [2, 9–11].

Currently, six categories of drugs are used in the treatment of BPH: herbal agents, selective *α*-adrenergic blockers, inhibitors of the enzyme 5 *α*-reductase, antimuscarinic agents, *β*3-adrenergic agonists, and, more recently, inhibitors of the enzyme phosphodiesterase type 5 [12–14]. Additionally, there are current evidences that nonsteroidal anti-inflammatory drugs (NSAID) can improve LUTS [15].

Herbal medicines are used in BPH therapy, and several mechanisms of action have been put forward based on the complex chemical composition present in plants. The presence of different substances acting on specific targets makes herbal medicines a relevant therapeutic strategy in the treatment of prostatic hyperplasia. The main herbal medicine used in the treatment of BPH is an extract of* Serenoa repens* fruit (Arecaceae), popularly known as “saw palmetto” for which there is strong evidence of clinical efficacy [16–18].

Many natural products are also used for the improvement of physiological functions as well as the symptoms of BPH. Among them, the pollen extract Cernitin (*Secale cereale*; Cernilton®),* Pygeum africanum* (Tadenan®),* Urtica dioica*,* Scutellaria baicalensis*,* Cucurbita pepo*, lycopene, and *β*-sitosterol are known for their beneficial effects on BPH [16–18]. Besides, many medicinal plant species have been tested* in vitro* and* in vivo* after they have shown potential for BHP treatment [19]. Some secondary metabolites from plants have also shown promising results* in vitro* and* in vivo*, among them phenolic substances such as isoflavones, lignans, and the stilbene resveratrol [20–24].

*Kalanchoe gastonis-bonnieri* Raym.-Hamet & H. Perrier (syn.* Kalanchoe adolphi-engleri* Raym.-Hamet) is a medicinal herb from the family Crassulaceae. It is used in Latin American medicine as a vaginal contraceptive as well as in the treatment of genital-urinary and vaginal infections [25]. In a previous study we reported the isolation of the new flavonoid quercetin 3-*O*-*α*-rhamnopyranoside-7-*O*-*β*-D-glucopyranosyl-(1→3)-*α*-L-rhamnopyranoside, as well as vicenin-2, a* C*-glycosyl flavone, from the leaf extract of KGB [26]. We also demonstrated that aqueous extracts from* K. gastonis-bonnieri* (KGB) are effective in controling dental bacterial plaque and calculus in dogs [27].

Extracts from KGB have been shown to immobilize, to clump together, and to promote structural changes in rat sperm [28]. The production and storage of seminal fluid and other components of semen are intrinsically linked to the functions of the prostate [29, 30]. Considering the ethnomedicinal use of KGB, this study aimed to determine the efficacy of this herb in the treatment of BPH, by using an* in vitro* model of BPH primary cell culture, particularly focusing on the main hallmarks related to the development of the disease.

## 2. Materials and Methods

### 2.1. Plant Material

In this study, leaves were collected from specimens in flower and from specimens not in flower of* Kalanchoe gastonis-bonnieri* cultivated in a residential garden in the city of Rio de Janeiro. Underground parts and flowers were also obtained from specimens growing in the same garden. A sample of a flowering specimen was identified and its voucher specimen (RGA 31592) is deposited in the Herbarium of the Botany Department at the Institute of Biology of the Federal University of Rio de Janeiro.

### 2.2. Extraction

Fresh leaves from* K. gastonis-bonnieri* (KGB) (average length of leaves: 15 cm) were rinsed with distilled water, cut into small pieces, and crushed in a blender. The extract obtained from the leaves of specimens not in flower was filtered and resulted in a clear yellow liquid. The color of the leaf extract of the specimens in flower was a salmon pink. The flowers were extracted by infusion with distilled water (20% w/w). The same procedure was applied for extracting the underground parts harvested from the other specimens in flower.  Table 1 shows the mass of the different parts of the* K. gastonis-bonnieri* specimens and the yield obtained from the extraction of each part. All the extracts were frozen, lyophilized, and kept in a freezer at -20°C.

### 2.3. HPLC-DAD/MS/MS

High-Performance Liquid Chromatography analyses with a Diode Array Detector coupled to a Tandem Mass Spectrometry (HPLC-DAD/ MS/MS) were carried out at the Center for Mass Spectrometry of Biomolecules-CEMBIO (IBCCF, UFRJ). The Prominence Shimadzu Liquid Chromatography system used was composed of an LC-20AD pump, a degasser system DGU-20A, and a DAD detector SPD-M20A, coupled to a Maxis Impact Q-TOF mass spectrometer (Bruker) equipped with an electrospray ionization (ESI) interface. An ODS-Hypersil reverse phase C-18 column (Thermo Scientific: 3 *μ*m, 150 mm, 2.1 mm) protected by a precolumn of the same material (3 *μ*m, 1 mm, and 2.1 mm) was used. The columns were maintained at 40°C.

A sample of the extract (4 mg/ml) from the underground parts of KGB was diluted in a mixture of water acidified with formic acid 0.1% and acetonitrile (19:1). The injection volume was 20 *μ*l.

The mobile phase consisted of eluent A, water containing 0.1% formic acid (Sigma-Aldrich), and eluent B, acetonitrile (Merck) containing 0.1% formic acid (Sigma-Aldrich). The samples were run for 40 min at 0.3 ml/min, and the absorbance was monitored between 210 and 400 nm. The gradient used was as follows: 0−5 min (5−20% B), 5−11 min (20−22% B), 11−12 min (22−100% B), 12−26 min (100% B), and 26−40 min (100−5% B).

The Q-TOF mass spectrometer was operated in the negative ion mode using the following parameters: capillary voltage, 5000 V; endplate offset, -500 V; pressure of nebulizer, 4 Bar; drying gas temperature, 200°C; Nitrogen was used as both the sheath and drying gas at a flow rate of 8.0 l/min. The mass range analyzed was set at m/z 50-1200 and collision energy at -5 eV. An external calibration solution (sodium formate 100 mM in water/isopropanol 1:1) was injected in the column and detected in the dead time ensuring mass accuracy throughout the chromatographic analysis. The elemental composition of the detected compounds was determined considering mass errors below 5 ppm. The data was processed using the Bruker Compass Data Analysis software.

### 2.4. BPH Cell

The stromal cell cultures were obtained from patients undergoing a clinical and histological diagnosis for BPH. BPH stromal cells were isolated according to previously described methods [31]. Briefly, prostate tissue was washed with phosphate-buffered saline (PBS) before being diced into approximately 1 mm^3^ pieces. The fragments were transferred to 10 ml dissociation flasks containing a solution of DMEM supplemented with 10% FBS and 1mg/ml of type I collagenase (Sigma, St Louis, MO). Tissue specimens were dissociated by constant stirring with a magnetic stir bar for 2-4 h at 37°C. The supernatant was frozen at 4°C and the remaining tissues were submitted to a new cycle of dissociation as described above. After that, the supernatants from the first and the second cycles were centrifuged and washed with balanced saline solution, without calcium and magnesium at 1200 RPM three times. The resulting cells were seeded in 25 mm^3^ flasks and left to allow attachment in a defined medium composed of supplemented DMEM (10% FBS, antibiotic/antimycotic mixture (Gibco): Penicillin 100 U/ml, Streptomycin 100 *μ*g/ml, and Fungizone 25 *μ*g/ml) and placed in a tissue culture incubator at 37°C in humidified air containing 5% CO_2_. Cells were fed 3 times a week. At subconfluence (approximately 90% occupancy in each bottle) they were harvested using 0.05% trypsin/EDTA (both from Sigma) and replated.

### 2.5. BPH Cell Proliferation Assay

The cell proliferation assay was performed using 1x104 BPH stromal cells per well in 96-well plates using Dulbecco's Modified Eagles Medium (DMEM) containing 0.5% ethanol and 1% Fetal Bovine Serum (FBS). Cells were treated with the extract of the underground parts, extract of leaves from the specimens in flower and not in flower, and extract of flowers of KGB for 72 hours. The treated BPH stromal cells and controls were washed once with PBS, fixed in a solution of 100% ethanol for 10 minutes, and then stained with 0.05% solution of crystal violet (Vetec) for 10 minutes. After staining, the cells were washed with distilled water and incubated in methanol for 5 minutes on a plate shaker, and the supernatant was collected. The absorbance was measured on an ELISA reader (iMARK BIO-RAD) at 570 nm.

In this assay, we evaluated the inhibitory effects of the four treatments (T1–T4) on cell proliferation of BPH stromal cells. The four treatments were the extract of the underground parts (T1), leaf extract of the flowering specimens (T2), extract of the flowers (T3), and leaf extract of the specimens not in flower (T4) at 250 *μ*g/ml for 72 hours.

### 2.6. BPH Cell Viability Assay

The cytotoxic potential of KGB underground parts extract (T1) against BPH stromal cells was assessed by a quantitative MTT colorimetric assay. This assay is based on the reduction of MTT by the mitochondrial enzyme NADH dehydrogenase tetrazolium dye in violet crystals known as formazan to detect and determine cell proliferation and viability [32]. The supernatants were removed from each well and replaced by the sample T1 in quadruplicate wells except for the zero time where after removing the supernatant 100 *μ*l MTT (3-[4,5-dimethylthiazol-2-yl]-2-5-diphenyltetrazolium bromide, 0.5 mg/ml, Sigma) was added. After addition of MTT, the culture plate was kept at 5% CO_2_ and temperature at 37°C for three hours. After this time, MTT was removed and 100 *μ*l of DMSO (Sigma) was added. The absorbance was read in an ELISA reader (BIO-RAD iMARK) at 570 nm. The same procedure was repeated 72 hours after the addition of the treatment and control.

The absorbance (optical density) of the treatments was calculated and their values were subtracted from the values for the wells incubated only with DMEM. Then, the percentage of cell viability was expressed using the formula: sample value (DMEM, DMEM + FCS or T1)/mean value at time zero (T = 0) x 100%.

### 2.7. BPH Cell Apoptosis Assay

After trypsinization and centrifugation, 1x10^5^ cells were resuspended in 200 *μ*l of propidium iodide solution (PBS, Triton X-100 0.1%, and propidium iodide 50 *μ*g/ml, Sigma) and incubated on ice for 5 minutes. After the incubation period, cell death was measured by flow cytometry (FACScalibur Becton Dickison) after acquiring 20,000 events. The excitation of the fluorochrome was measured using an argon laser with a wavelength of 488 nm and the emission was collected through a filter 630/22 nm.

### 2.8. Statistical Methods

All data represent the mean ± standard deviation values of three independent experiments. Differences between groups were analyzed using one-way ANOVA followed by the multiple comparison Newman-Keuls test. The value p <0.05 (*∗*) was considered statistically significant.

## 3. Results and Discussion

Four aqueous extracts were prepared from leaves, flowers, and underground parts of* K. gastonis-bonnieri* (KGB) specimens. The yield of the extract from leaves collected from the specimens not in flower (2.1 %) was similar to that observed for leaves from flowering specimens (1.9 %), while the yields from the underground parts from flowering specimens and flowers were 1.2 % and 3.9 %, respectively (Table 1).

**Figure 1:**
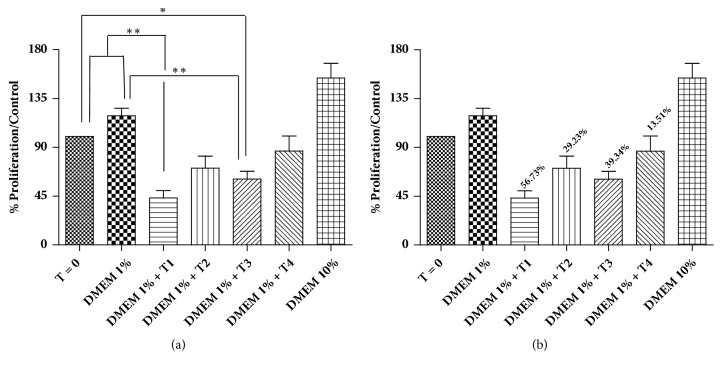
Proliferation of BPH stromal cells treated with different extracts of* Kalanchoe gastonis-bonnieri* (KGB). (a) Graph representing the proliferation of cells with the different extracts (T1-T4) of KGB at 250 *μ*g / ml after 72 h. *∗* p<0.05, *∗∗* p<0.001. Data represent the mean ± standard deviation values of three independent experiments. (b) Percentage of BPH stromal cells after treatment with four different extracts (T1-T4) of KGB. Data represent the mean ± standard deviation values of three independent experiments.

We evaluated the inhibitory effects of the four KGB preparations on the proliferation of stromal cells from primary benign prostatic hyperplasia (PBH). Underground parts extract from the flowering specimens of KGB (T1 treatment), leaf extract of KGB from flowering specimens (T2 treatment), flowers extract from KGB (T3 treatment), and leaf extract of KGB not in flower (T4 treatment) were tested at 250 *μ*g/ml for 72 hours. T1, T2, T3, and T4 treatments inhibited the cells proliferation by 56.7%, 29.2%, 39.4%, and 13.5%, respectively (Figure 1).

The best results were observed for the underground parts extract (T1 treatment), which encouraged the continuation of our experiments with this sample in order to corroborate the preliminary detection of its activity in BPH.

### 3.1. BPH Cell Viability Assay

**Figure 2:**
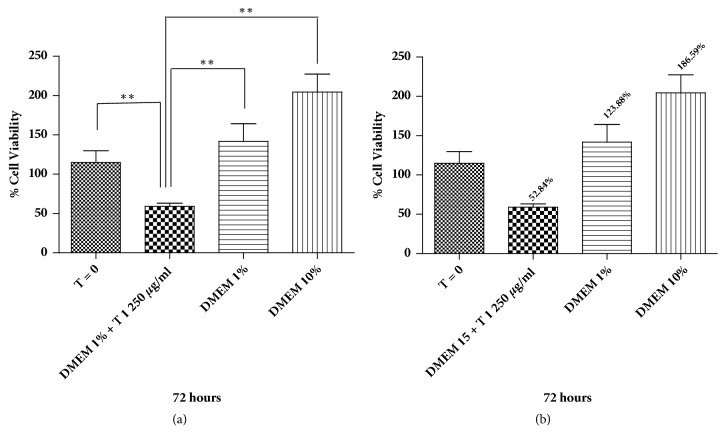
Cell viability assessed by MTT. (a) Graph representing the viability of BPH stromal cells with the underground parts extract of KGB (T1 treatment) at the concentration of 250 *μ*g/ml. *∗* p<0.05, *∗∗* p<0.001. Data represent the mean ± standard deviation values of three independent experiments. (b) Percentage of BPH stromal cells after treatment with extract of KGB underground parts (T1 treatment). Data represent the mean ± standard deviation values of three independent experiments.

The extract of KGB underground parts (T1 treatment) significantly reduced the viability of BPH stromal cells treated with 250 *μ*g/ml, promoting decay of more than 50% cell viability (Figure 2).

### 3.2. BPH Cell Apoptosis Assay

In order to clarify whether the reduction in the number of BPH stromal cells observed after the treatment with KGB underground parts was due to a blockage in the proliferation activity of these cells or due to an induction of cell death, we performed an apoptosis assay to address this question.

**Figure 3:**
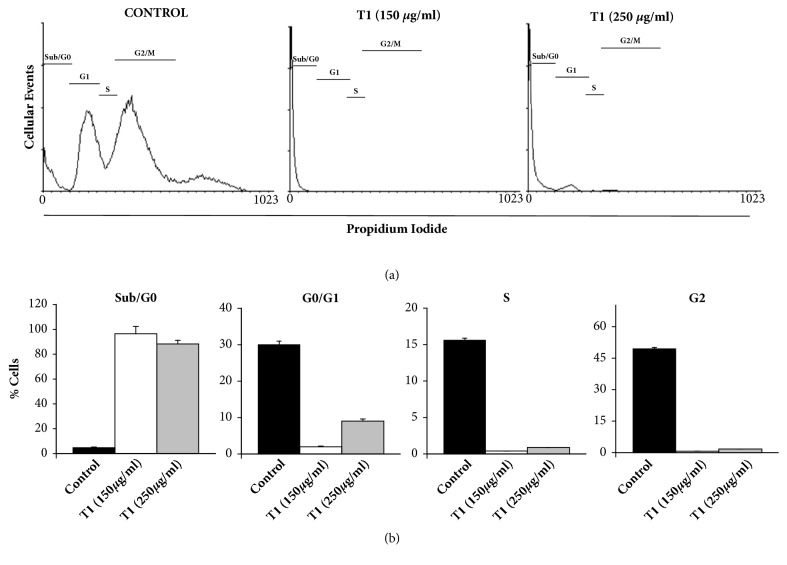
BPH cell death evaluation by flow cytometry. (a) Histogram representing the distribution of BPH stromal cells in different phases of the cell cycle after treatment with the underground parts extract of* Kalanchoe gastonis-bonnieri* (T1) at concentrations of 150 *μ*g/ml and 250 *μ*g/ml after 72 h. *∗* (p <0.001). Data represent the mean ± standard deviation values of three independent experiments. (b) Percentage of BPH stromal cells in different phases of the cell cycle after treatment with the underground parts extract of* Kalanchoe gastonis-bonnieri* (T1) at concentrations of 150 *μ*g/ml and 250 *μ*g/ml after 72 h. *∗* (p <0.001). Data represent the mean ± standard deviation values of three independent experiments.

The percentage of dead cells detected in the sub/G0 region of the cell cycle after 72 hours was significantly higher when the cells were seeded in the presence of T1 treatment in the concentrations of 150 *μ*g/ml and 250 *μ*g/ml (Figure 3(a)).

However, the activity was not dependent on the concentration used. We observed that both concentrations of the extract were able to suppress the progression of BPH cells along the cell cycle, with no significant difference between them. Additionally, Figure 3(b) shows that there was, in the control group, a distribution throughout the different cell cycle phases, including the S and G2/M phases, thus indicating a proliferating profile of the BPH stromal cells in the absence of the KGB treatment. On the other hand, the treatment with T1 showed that BPH stromal cells were almost exclusively at the sub/G0 phase of the cell cycle.

Despite numerous reports on the use of plants or derivatives of natural products of plant origin for the treatment of benign prostatic hyperplasia, these activities have not always been proven in pharmacological studies.

The inhibitory activity observed for KGB in BPH cell proliferation was very effective, since the KGB underground parts (T1) at the concentration of 150 *μ*g/mL was able to drastically reduce the proliferation activity and the viability of BPH stromal cells in 72 hours. Moreover, the same treatment also induced a strong increase in the apoptosis rates of BPH stromal cells, since a large percent of these cells were restricted in the sub/Go phase of the cell cycle, as revealed by the flow cytometry analysis. In addition, the cell cycle profile presented by BPH stromal cells after the treatment with the KGB underground parts corroborated the proliferation data, as the normal transition throughout the cell cycle was blocked by the KGB treatment. Therefore, these results that show a concomitant decrease in the cell viability and proliferation, combined with an induction of cell death by apoptosis, may reveal a beneficial role of KGB in combating the process of prostate growth that culminates in the development of BPH.

Our results with KGB underground parts are comparable with those observed for extracts from two plants clinically used to treat BHP. The first one,* Pygeum africanum *(Tadenan®), inhibits the proliferation of cultured human prostatic myofibroblasts and fibroblasts as well as enhances apoptosis at concentrations from 25 to 100 *μ*g/ml [33, 34].* Serenoa repens* (Permixon®), the second one, revealed a tissue-selective action resulting in morphological changes and augmented apoptosis rates in addition to the inhibition of nuclear membrane bound 5*α*-reductase isoenzymes catalytic activity in prostate cells at the concentration of 10 *μ*g/ml [35]. Additionally, an increment of Bax-to-Bcl-2 expression and caspase 3 activity, molecules involved in the apoptotic pathway, has already been documented in prostatic tissue samples from BPH symptomatic patients under treatment with Permixon for at least 3 months [36]. Also, an* in vitro* study showed that treatment with* S. repens* leads to the lightening of BPH symptoms due to antiproliferative and proapoptotic effects exerted on prostate epithelia and triggered by the downregulation of IGF-1 signaling pathway and induction of JNK [37]. Finally, BPH treatment with finasteride, the main therapeutic approach employed for this disease management, also demonstrated a mechanism exclusively observed in epithelial cells: caspase-dependent apoptosis initiation through activation of caspases 3 and 6 [38]. In fact, the success of the main treatment approaches (phytotherapic or conventional pharmacologic drugs) that are routinely used in the treatment of BPH are related to the control of BPH growth by reducing proliferation and inducing apoptosis [33, 34, 36]. Thus, the effects of KGB on BPH seem very interesting, since this disease is largely characterized by an imbalance between the proliferation and apoptosis [4, 7, 8, 11]. Furthermore, it was recently shown that KGB underground parts were able to abrogate the androgen signaling in prostate malignant cell lineages, besides inducing the apoptosis via caspase 8 activation, thus reinforcing the therapeutic potential of KGB in prostatic diseases [39].

Although the greater activity is in the subterranean parts from* K.* gastonis-*bonnieri* and therefore could be a disadvantage for a phytomedicinal preparation due to the nonrenewable characteristics of this part of the plant, this succulent herb is a fast growing-species that propagates easily by asexual reproduction [40].

### 3.3. Chemical Composition of KGB Underground Parts

**Figure 4:**
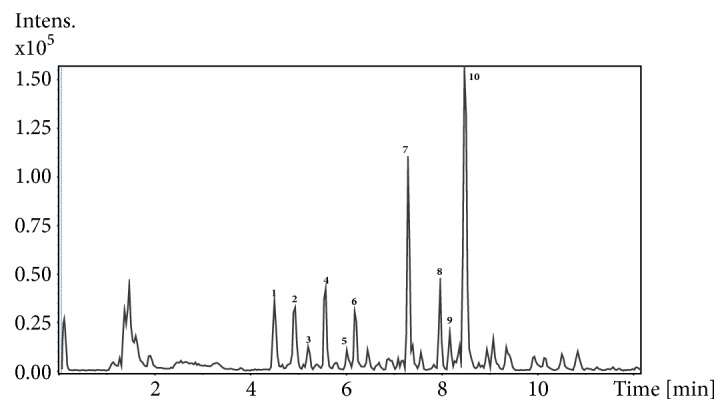
Base peak chromatogram (LC-MS) of aqueous extract from KGB underground parts (4 mg/ml) in the negative ion mode.

The extract from the KGB underground parts had its chemical composition assessed by HPLC-DAD/MS/MS in the negative ion mode. As the TOF analyzer enables high-resolution mass measurements, with mass errors below 5 ppm, it was possible to infer the molecular formula of the major constituents detected in the KGB underground parts. The resulting chromatogram is shown in Figure 4, while data on the major compounds detected are summarized in Table 2.

Peak 1 (Rt 4.5 min; *λ*max 261 nm) presented the [M-H]^−^ ion at m/z 359.0994 (C_15_H_19_O_10_) as base peak. MS/MS spectrum showed a fragment at m/z 197.0458 (C_9_H_9_O_5_), suggesting the loss of a hexose unity. This substance could possibly correspond to a glycosylated form of syringic acid such as syringate 4-*O*-*β*-glucopyranoside [41]. However, isomers of syringic acid cannot be ruled out. Peak 3 (Rt 5.2 min; *λ*max 282 nm) also showed a [M-H]^−^ ion for which the molecular formula C_15_H_19_O_10_ was proposed and a similar fragment at m/z 197.0457. We hypothesize that this substance could correspond to a glycosyl ester of syringic acid. Syringic acid *β*-D-glucopyranosyl ester has already been reported for leaves of* Kalanchoe pinnata* and a derivative of this substance was recently reported in the underground parts of the same species [42, 43]. Thus, peak 1 could correspond to a syringic acid glycosylated at the phenolic hydroxyl, having a free carboxyl moiety, and peak 3 to the same aglycone glycosylated at the carboxyl moiety. This is corroborated by their UV spectra, which correspond to those of the aforementioned substances and the order of elution, since an ester is less polar than a carboxylic acid.

Peak 6 (Rt 6.2 min) showed the [M-H]^−^ ion at m/z 381.1780 (C_16_H_21_O_9_), with fragments at m/z 235.1196 (C_10_H_19_O_6_) and 161.0458 (C_6_H_9_O_5_) at the MS/MS spectrum, corresponding to the loss of a deoxyhexose (e.g., rhamnose) and a C_4_H_10_O (butanol) unity, respectively. Peak 7 (Rt 7.3 min) in its turn presented the [M-H]^−^ ion at m/z 415.1618 (C_19_H_27_O_10_) and fragments at m/z 269.1037 (C_13_H_17_O_6_) and 161.0456 (C_6_H_9_O_5_), corresponding to the loss of a deoxyhexose moiety and a C_7_H_8_O (benzyl alcohol) unity. Peak 8 (Rt 8.0 min) gave a [M-H]^−^ ion at m/z 395.1935 and fragments at m/z 249.1352 (C_11_H_21_O_6_) and 161.0461 (C_6_H_9_O_5_), corresponding again to a loss of a deoxyhexose and a C_5_H_12_O (pentanol or methylbutanol) unity. Thus, peaks 6, 7, and 8 were tentatively attributed to glycosidically bound volatile substances, which are commonly found in plants [44–46]. Alcohol and monoterpene glycosides are found in the roots of plants from* Rhodiola* species, which also belong to the Crassulaceae family [47].

Peaks 9 (Rt 8.2 min) and 10 (Rt 8.5 min) presented the [M-H]^−^ ions at m/z 521.2022 and 521.2035, respectively. Both presented C_26_H_33_O_11_ as molecular formula [M-H]^−^ and a MS/MS fragment at m/z 359.1508, indicative of the loss of a hexose unity. As we did not observe any further fragmentation of the aglycones, many structural possibilities were found for these substances. All the possible substances of natural origin with this molecular formula found in the Sci-Finder database belonged to the class of lignans, with several possible skeletons. Thus, we postulate that peaks 9 and 10 correspond to glycosylated lignans. In the underground parts of* Kalanchoe pinnata*, a glycosylated aryltetralin lignan was recently reported [43]. Lignans are also present in roots of* Rhodiola* species [48].

There are several reports on the activity of lignans in BPH and prostate cancer. For instance, a lignan-enriched extract from flaxseed (Beneflax®) was capable of improving LUTS in patients with BPH in a double-blind placebo-controlled clinical trial [23]. A similar extract from flax hulls prevented the development of testosterone propionate- (TP-) induced BPH in rats [49]. Also, secoisolariciresinol diglucoside, the major lignan in flaxseed, was able to inhibit BPH in TP-induced BPH in rats. Enterolactone, a metabolite of this substance, was shown to block the proliferation of a human prostatic stromal cell line by a mechanism involving the G protein-coupled estrogen receptor 1 [50]. Furthermore, the lignans from the medicinal species* Campylotropis hirtella* (Fabaceae) were shown to inhibit prostate specific antigen and to decrease the androgen receptor expression in a prostate cancer cell linage. The most potent of those lignans (dehydrodiconiferyl alcohol) was further investigated and exhibited proapoptotic effects in these cells [24].

It was not possible to identify peaks 2 (Rt 4.9 min), 4 (Rt 5.5 min), and 5 (Rt 6.0 min). We reported the molecular formulas considered most likely here, with the smallest possible errors. However, we do not discard other structural possibilities for these peaks.

## 4. Conclusion

The present results seem very encouraging, since they reveal a potential use of the underground parts of* Kalanchoe gastonis-bonnieri* in the treatment of benign prostatic hyperplasia, a condition that causes significant chronic morbidity for men. Furthermore, the increment in the “phytotherapic products portfolio” currently available could improve the management of this disease, since a large number of natural compounds has been described as reliable, safe, and cost effective in the treatment of several diseases.

In addition, the main mechanisms related to KGB treatment seem to be the inhibition of the proliferation activity along with the induction of apoptosis.

## Figures and Tables

**Table 1:** Extraction and yield of different parts of *K. gastonis-bonnieri*.

**Fresh plant material/mass (g)**	**Lyophilized ** **Extract (g)**	**Yield** %
Leaves of plants not in flower (1139.2)	23.8	2.1
Leaves of plants in flower (149.5)	2.9	1.9
Underground parts of plants in flower (82.9)	0.8	1.2
Flowers (87.3)	3.4	3.9

**Table 2:** Major chemical compounds in the extract of underground parts from *K. gastonis-bonnieri* by HPLC-DAD/MS/MS.

**Peak No.**	**Rt (min)**	**Molecular formula [M-H]** ^−^	**Measured m/z [M-H]** ^−^	**Calculated [M-H]** ^−^	**Error (ppm)**	**UV** ***λ*** **max (nm)**	**MS/MS fragment ions**	**Proposed compound**
1	4.5	C_15_H_19_O_10_	359.0986	359.0984	-0.6	261	197.0457	Syringic acid hexoside
2	4.9	C_12_H_20_NO_8_	306.1194	306.1194	0	n.d.	205.0380; 161.0455	Unknown
3	5.2	C_15_H_19_O_10_	359.0996	359.0984	-3.6	282	197.0458; 239.0572	Syringic acid hexoside
4	5.5	C_18_H_24_N_5_O_6_	406.1732	406.1721	0.1	255	307.1040	Unknown
5	6.0	C_16_H_21_O_9_	357.1196	357.1191	-1.3	273	177.0556	Unknown
6	6.2	C_16_H_29_O_10_	381.1780	381.1766	-3.5	n.d.	235.1196; 161.0458	Alkyl diglycoside
7	7.3	C_19_H_27_O_10_	415.1618	415.1610	-1.9	n.d.	269.1037; 161.0456	Benzyl diglycoside
8	8.0	C_17_H_31_O_10_	395.1935	395.1923	-3	n.d.	249.1352; 161.0461	Alkyl diglycoside
9	8.2	C_26_H_33_O_11_	521.2035	521.2028	-1.2	n.d.	359.1507	Glycosylated lignan
10	8.5	C_26_H_33_O_11_	521.2022	521.2028	1.3	283	359.1508	Glycosylated lignan

## Data Availability

The data used to support the findings of this study are included within the article.
